# Humanitarian Training With Virtual Simulation During a Pandemic

**DOI:** 10.1017/dmp.2021.152

**Published:** 2021-05-19

**Authors:** Sean M. Kivlehan, Kathryne Tenney, Sam Plasmati, Vincenzo Bollettino, Katie Farineau, Eric J. Nilles, Greg Gottlieb, Stephanie R. Kayden

**Affiliations:** 1 Department of Emergency Medicine, Brigham and Women’s Hospital, Boston, MA, USA; 2 Harvard Humanitarian Initiative, Cambridge, MA, USA; 3 Harvard School of Public Health, Boston, MA, USA; 4 Feinstein Institute, Tufts University, Boston, MA, USA

**Keywords:** disasters, professional education, emergencies, relief work, simulation training

## Abstract

There is an ongoing and established need for humanitarian training and professionalization. The coronavirus disease 2019 (COVID-19) pandemic disrupted training programs designed to accomplish this goal, including the Humanitarian Response Intensive Course, which includes a 3-d immersive simulation to prepare humanitarian workers for future field work. To provide program continuity, the 3-d simulation was quickly adapted to a virtual format using a combination of video conferencing, short messaging service, and cloud-based file storage software. Participants were geographically dispersed and participated virtually. Learning objectives were preserved, while some components not amenable to a virtual format were removed.

A virtual humanitarian training simulation is a feasible, acceptable, and affordable alternative to an in-person simulation. Participants were engaged and experienced minimal technological disruptions. The majority of students believed the format met or exceeded expectations. However, feedback also emphasized the importance of providing sufficient time for team collaboration and deliverable preparation in the simulation schedule. The virtual format was more affordable than the traditional in-person simulation, and diverse expert faculty who could not have attended in-person were able to participate. This format could be used to overcome other barriers to in-person simulation training, including geographic, financial, time, or security.

An estimated 168 million people are in need of humanitarian assistance around the world, a number that has steadily risen over past years.^1^ Entering 2020, there were active response plans in 53 countries with an overall funding gap of 46%.^1^ The emerging coronavirus disease 2019 (COVID-19) pandemic continues to exacerbate this already challenging situation by further increasing demands on health care and finances.^2^ Compounding this is a disruption of the humanitarian workforce by pandemic-related travel restrictions and economic challenges.^3^ Formal training programs for humanitarian workers, designed to improve the professionalism and effectiveness of a response, have been canceled. However, the need for humanitarian support marches on, accelerated by the pandemic. We describe the rapid transition of an in-person humanitarian simulation training program to an online venue in response to the challenges brought by COVID-19 and propose its broader utility in overcoming barriers to humanitarian training.

The Humanitarian Response Simulation Exercise is a 3-d simulation hosted annually by the Harvard Humanitarian Initiative (HHI) for approximately 120 graduate students and humanitarian professionals in a state park in Massachusetts.^4^ The simulation exercise is the combined culmination of 2 courses: a 60-student graduate course on humanitarian response at the Harvard T. H. Chan School of Public Health and a 60-student professional course called the Humanitarian Response Intensive Course (HRIC). The students act as responders from real-world humanitarian organizations and are tasked with conducting a rapid assessment of a complex humanitarian emergency and preparing a service delivery plan outlining their organization’s proposed response. Students spend the entire simulation in role, interacting with roughly 130 volunteer actors portraying beneficiaries, members of United Nations (UN) agencies and nongovernmental organizations (NGOs), and both state and nonstate armed forces. During the simulation each NGO team partakes in hands-on humanitarian training activities between needs assessments and stakeholder interviews, all while managing frequent stressful surprise incidents. Since its inception in 2004, the Simulation Exercise has trained more than 800 participants from more than 85 countries, representing over 200 NGO, government, and other organizations.^5^

There is an established need for professionalism and training in the humanitarian sector.^6–8^ Several reforms to the international humanitarian system, such as the creation of the UN Office for the Coordination of Humanitarian Affairs (OCHA), the cluster approach, the SPHERE project, and the Core Humanitarian Standards, have codified standards, ethics, and practices.^9–12^ Many institutions now offer humanitarian-focused curricula, certificates, concentrations, and degrees to train current and future aid workers in these standards.^7^ Humanitarian staff should be trained in an ethical way that safely exposes them to realities of the field and increases competency in difficult working environments. Humanitarian simulation exercises have evolved as a solution to provide a safe stress environment, but there continue to be barriers to accessing these trainings, including cost, travel, and language.^7,13^

The abrupt onset of the COVID-19 pandemic and subsequent restrictions to travel and in-person gatherings exacerbated these barriers. The professional HRIC course was canceled due to COVID-19, but the graduate course continued and transitioned to a virtual setting along with all other courses at the School of Public Health. Instead of canceling the simulation, the decision was made to convert it to a virtual setting for the 60 graduate students. This manuscript shares the experience and reception of the format conversion while exploring the challenges of virtual humanitarian simulation training.

## Methods

A working group of simulation faculty, technological experts, simulation alumni, and HHI faculty with experience directing another virtual humanitarian training program, the National NGO Program on Humanitarian Leadership (NNPHL), was established.^14^ The experiences of other virtual training simulations were reviewed through both a literature review and personal outreach by the author group. While online humanitarian training has grown substantially over the past decade, this has been met with both skepticism and optimism.^15^ Many online programs suffer from low engagement and lack of a clear instructional strategy.^16–19^ Hands-off skills training could be less effective as the tactile and muscle memory does not occur.

The content of the in-person 3D simulation was reviewed in detail, which has a continuous 52-h timeline. It was decided that the simulation would be condensed to a single day 9-h activity to allow for easier scheduling. While this change restricted the quantity of content, the virtual simulation would only have 60 students instead of the standard 120. Zoom (Zoom Video Communications, Inc, San Jose, CA, USA) was used for the video conferencing platform as it was already the platform for the students’ other coursework.

Faculty, volunteers, and alumni were polled for suggestions on how to preserve the simulation’s learning objectives and key experiences, which include immersion in stressful environment, team building, interaction with diverse role-playing stakeholders, and surprise crises. A desire was expressed to maintain a mixed linear and open-ended learning environment. Some in-person events were excluded after review, such as a simulated abduction and militia-led interruptions, as they would not translate meaningfully to the virtual environment. Elements designed to improve student engagement we included, such as frequent small group meetings with country directors and frequent deliverable requirements. Ultimately, key learning objectives were preserved, and the virtual setting allowed for recruitment of highly qualified faculty from around the world who otherwise would not have been able to participate.

Students were divided into 9 teams to allow students to experience and learn from the benefits and challenges of working in small groups. Each team was built to ensure internal diversity with regard to prior experience and other factors, and each team had a pre-identified leader and information manager. Teams were expected to be in-role for the entire simulation and were each assigned a faculty member as their country director, an in-role position developed to guide, transmit, and receive information and deliverables to and from the teams. The country director stayed in touch with each team leader by means of WhatsApp (Facebook, Inc, Menlo Park, CA, USA) throughout the simulation. Simulation leadership, who were out-of-role, communicated with teams only by means of the country directors, who stayed in-role. There was an out-of-role WhatsApp channel open to all for technological support as needed, and several in-role channels were created (Table 1). Dropbox (Dropbox, Inc, San Francisco, CA, USA) files were created for each team where new documents were deposited and deliverables were returned; the team leader, information manager, and country director were given access. Volunteer role players received overviews detailing their roles, scripts, and schedules and were encouraged to dress to their part and create simulated backgrounds representing their settings.

**Table 1.** WhatsApp channels

• All Participants • All Students • All Faculty & Volunteers • Simulation Coordinators and Leadership • All Country Directors • All Cluster Leads • All Team Leaders • Each Team + Country Director • Technical Support Channel

**Figure 1.**
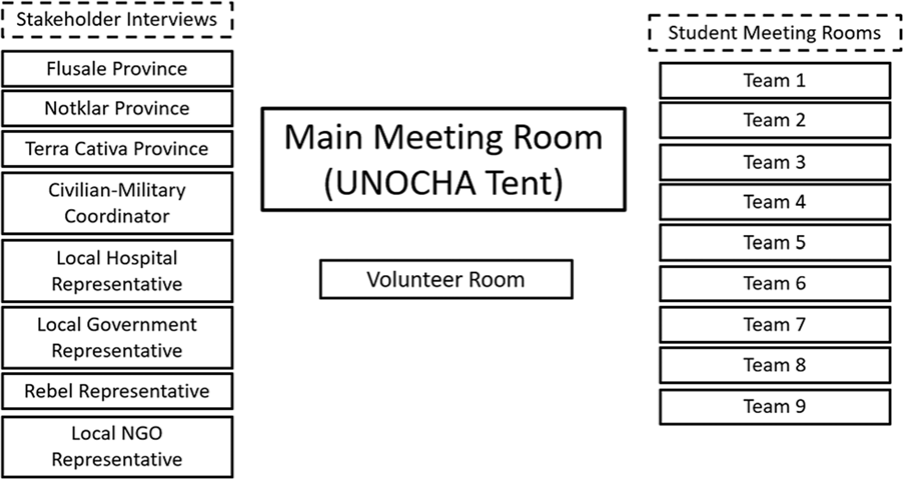
Virtual simulation rooms.

Each day of the in-person simulation was condensed into an hour for the virtual simulation. Each of these hour-long “days” was called a round. Each round was initiated with an updated situation report on how the scenario was evolving and culminated with a team-based deliverable due to the team’s country directors at the conclusion of the round. A general room was open throughout the simulation, which served as a staging area for breakout rooms and as the venue for simulation-wide meetings in between each day/round and for the initial and final simulation segments. The overall schedule is shown in Table 2, and the virtual room layout is shown in Figure 1.

**Table 2.** Simulation schedule

**Orientation**	**9:00 - 10:00 AM**
• Description of Sim • Intro Role Players • At 9:30, teams break off to prepare Mission Statement, role in Worani, and fill out passport to share with CD at 9:50	
**Round 1 [Day 1]**	**10:00 AM - 11:00 AM**
• Goal is to prepare AMENDS analysis • Get Intro to round and receive SitRep 1 • Each team has a scheduled 10 min interview with 1 of 3 IDP/refugees • Other than that, they freely speak to the other role players • By 11:00, they need to submit AMENDS analysis to CD	
**Round 2 [Day 2]**	**11:00 AM - 12:15 PM**
• Goal is to begin FSDP and prepare for Cluster Meeting • Get Intro to round and receive SitRep 2 • Each team has a scheduled 10 min interview with 1 of 3 IDP/refugees • Team can speak freely to other role players while prepping for Cluster Meeting and starting FSDP • Reconvene in main Zoom room at 11:45 AM • Cluster Meeting at 11:55 AM	
**Working lunch - Spend time working on FSDP**	**12:15 - 1:15 PM**
**Round 3 [Security Incident/Media Incident]**	**1:15 - 2:15**
• Round 3 starts with 10 min Team Leader Meeting to coordinate overall FSDPs • During that meeting, someone else on each team receives a Security Incident SitRep (3 different scenarios - kidnapping (SIR), key informant is accused of human trafficking (SIR), local newspaper accuses org of embezzlement (Press)) • Teams thought they had this time to work on FSDP, instead they must create Security Incident Report or Press Release • Report is due to CD at 1:45 PM • Reconvene in main tent at 1:55 PM • 2:00 - 2:15 PM - 3 teams called at random for impromptu press conference	
**Final prep**	**2:15 - 3:00 PM**
• At 2:45 PM - team leaders are “recalled to the capital” and cannot present during FSDP - they need to leave group to attend 15 min pointless meeting with govt rep • Unannounced VIP meeting	
**FSDP**	**3:00 - 4:00 PM**
• 3 min per team presentation • 2 min per team questions	
**Debrief**	**4:00 - 5:00 PM**

Abbreviations: CD, Country Director; FSDP, Final Service Delivery Plan; SIR, Security Incident Report; VIP, Very Important Person.

During each day/round, several beneficiaries and stakeholders could be interviewed to gather information; each could be found in a different communities or locations represented by predefined breakout rooms. Teams were provided with a list of potential communities to visit in addition to appointments with specific beneficiaries and stakeholders to provide a linear framework and balance crowding while preserving the open-ended nature of the simulation.

Student performance was monitored in real time by volunteer role players. Simulation leadership monitored the rooms to observe the experience and provided out-of-role feedback and guidance in real time to country directors, who could then relay this to teams in-role. Feedback from faculty and volunteers on student performance on the individual and team level against the course learning objectives was collected.

The simulation concluded with an hour-long debrief to allow for 360-degree feedback from all participants; the standard course evaluation form was used. Anonymous feedback from students was solicited after course completion on a 5-point Likert scale.

## Results

Student feedback is summarized in Table 3. Responses were positive, with a majority responding either “good” or “very good” to nearly all questions. The final service delivery plan received notable negative feedback, which comments attributed to a lack of sufficient preparation time during the simulation. The responses to “Simulation met its stated objectives” and “Experience working in the simulated cluster system” included an increased number of negative responses as well, again with comments suggesting that additional preparation and collaboration time was needed.

**Table 3.** Student feedback

Question	Very poor	Poor	Neutral	Good	Very good
Simulation met your expectations	1 (5%)	2 (10%)	4 (20%)	12 (60%)	1 (5%)
Simulation met its stated objectives	0	4 (20%)	6 (30%)	7 (35%)	3 (15%)
Experience with the following role players:					
Beneficiaries	0	0	2 (10%)	9 (45%)	9 (45%)
Country Directors	0	2 (10%)	4 (20%)	6 (30%)	8 (40%)
Cluster Leads	0	0	8 (40%)	5 (25%)	7 (35%)
General Role Players	0	0	3 (15%)	9 (45%)	8 (40%)
Experience working in the simulated cluster system	1 (5%)	1 (5%)	6 (30%)	7 (35%)	5 (25%)
Experience with the following deliverables:					
NGO Mission Statement and Passports	0	4 (20%)	5 (25%)	6 (30%)	5 (25%)
AMENDS Analysis	0	2 (10%)	5 (25%)	8 (40%)	5 (25%)
Security Incident Report or Press Report	0	2 (10%)	4 (20%)	6 (30%)	8 (40%)
Final Service Delivery Plan	0	7 (35%)	4 (20%)	3 (15%)	6 (30%)
Overall Administration and Logistics of the Simulation	0	1 (5%)	3 (15%)	9 (45%)	7 (35%)

In the course survey, students reported experiencing stress throughout the day due to time pressures, surprise tasks, and deliverable deadlines. The consensus was that, despite minor technological issues throughout the day, the simulation environment was successfully created and maintained.

## Discussion

Students and faculty were dispersed globally and participated synchronously. Zoom was an effective platform for the simulation. Some participants reported challenges operating multiple platforms due to personal computer processing limitations, and a small number reported occasional bandwidth challenges.

The country director role was critical to the success of the virtual simulation as they served as the central in-role communication point for student NGO teams, coordinators, and role players. This allowed for students to be ushered throughout the simulation without breaking role, and for the country directors to serve as a bidirectional communication access between students in role and coordinators out of role.

Establishing a short messaging service (SMS) communication channel was crucial for both intra- and inter-team communication, as well as simulation-wide communication and prompting. Using a cloud-based file storage service allowed for real-time collaborative editing and sharing of documents and deliverables. In this simulation, WhatsApp and Dropbox were used; however, other platforms may be as or more effective.

The reduction of team collaboration and deliverable preparation time can be attributed to the loss of the evening down time that teams had in the full in-person simulation. Future virtual simulations should include protected down time for team collaboration. The condensed time frame made meeting deadlines difficult as there was little protected time for team deliverable preparation. Defined break periods are important for a full day in front of a computer. Time zone differences should be considered during planning of synchronous learning experiences when participants are broadly dispersed.

These results are limited by a lack of participation, as only 20 (37%) of participants completed the evaluation. This low response rate may have been due to the timing of the evaluation, which was launched several days after course completion. Reminder messages were sent to improve the response rate, but it remained poor. This may be due to student graduations and email changes, as the simulation was held at the end of the school year and many students who participated graduated immediately after.

The simulation allowed for many participants to take unanticipated leadership roles. Planned and unplanned events created opportunities for pressure and stress, which are important learning components of simulations.^20^ Students and faculty reported the virtual simulation to be a useful exercise that improved their knowledge and confidence.

## Conclusions

This real-world experience of transitioning a well-established and long-running humanitarian simulation exercise into a virtual simulation revealed challenges and opportunities for future humanitarian training. Despite some limitations, the virtual platform provides advantages over an in-person setting, including the ability to recruit a more diverse faculty. Feedback emphasized the importance of providing sufficient time for team collaboration and deliverable preparation in the simulation schedule. Virtual humanitarian simulation training could be used for program continuity when travel is restricted not only due to a pandemic, but also due to financial, time, or security concerns.
